# Accuracy of Patient Identification of Electrocardiogram-Verified Atrial Arrhythmias

**DOI:** 10.1001/jamanetworkopen.2020.5431

**Published:** 2020-05-21

**Authors:** Jeffrey L. Turner, Ann Lyons, Rashmee U. Shah, Brian Zenger, Rachel Hess, Benjamin A. Steinberg

**Affiliations:** 1 Division of Cardiovascular Medicine, University of Utah Health Sciences Center, University of Utah, Salt Lake City; 2 Data Science Services, University of Utah Health Sciences Center, Salt Lake City; 3 Department of Population Health Sciences, University of Utah, Salt Lake City

## Abstract

This cross-sectional study describes the sensitivity and specificity of patient self-assessment for atrial arrhythmia compared with 12-lead electrocardiogram and describes the association of patient perception of arrhythmia with symptom burden.

## Introduction

Atrial fibrillation (AF) is the most common arrhythmia in adults, and treatment of AF is often focused on improving symptoms. Patient-reported outcomes can provide standardized, health-related, quality-of-life end points to guide and support clinical decisions. The association of patient symptoms with arrhythmia may be confounded. We sought to understand the accuracy of patients’ identification of their arrhythmia and association with perceived symptom burden. Specifically, the objectives of this cross-sectional study were to describe the sensitivity and specificity of patient self-assessment for atrial arrhythmia compared with 12-lead electrocardiogram (ECG) and to describe the association of patient perception of arrhythmia with symptom burden.

## Methods

This study was approved by the University of Utah institutional review board. A waiver of consent was granted because the study used anonymous, aggregate data that were collected as part of routine clinical care.

All patients with AF in our electrophysiology clinic are asked to complete the Toronto Atrial Fibrillation Severity Scale (AFSS) patient-reported outcomes assessment immediately before the clinic visit.^1^ The details of our system have been described previously.^2,3^ Among its 4 domains, the AFSS requests that patients identify their current rhythm; it also yields a 35-point symptom score (referred to hereafter as the AFSS symptom score), with higher scores reflecting increasing patient perceived AF symptom burden (range, 0-35 points). We compared single-visit patient self-awareness results with the results of same-day, in-clinic, 12-lead ECG (reference standard) to understand patient accuracy of atrial arrhythmia self-identification. We also assessed perceived vs actual atrial arrhythmia compared with AFSS symptom score.

Categorical variables are summarized as number (percentage), and continuous variables are summarized as mean (SD). Univariate comparisons were performed with χ^2^ tests for categorical variables and analysis of variance for continuous variables. Two-sided *P* < .05 was considered statistically significant. All analyses were performed using R statistical software version 3.5.2 and RStudio version 1.2.1335 (both from R Project for Statistical Computing), with specialty packages. Data analysis was performed from September 2018 to September 2019.

## Results

From October 2016 to February 2019, 656 patients (mean [SD] age, 66.34 [11.94] y; 255 women [38.9%]) responded to the AFSS question regarding current atrial rhythm and had an interpretable ECG from the same day. Baseline characteristics of these patients, stratified by ECG rhythm and response, are shown in the Table. Most of the patients were white (599 patients [91.3%]), 75.5% (495 patients) had a history of hypertension, and 38.3% (25 patients) were taking a β-blocker. The mean (SD) left ventricular ejection fraction was 59.36 (10.11).

**Table.** Baseline Characteristics Overall and Stratified by Rhythm and Identification^a^

Characteristic	Patients, No. (%)					*P* value^b^
	Overall (N = 656)	AT/AF on ECG				
		Yes		No		
		Correctly identified (n = 102)	Incorrectly identified (n = 58)	Correctly identified (n = 453)	Incorrectly identified (n = 43)	
Age, mean (SD), y	66.34 (11.94)	69.32 (9.53)	72.22 (7.93)	64.91 (12.39)	66.49 (13.29)	<.001
Age <65 y	257 (39.2)	35 (34.3)	6 (10.3)	199 (43.9)	17 (39.5)	<.001
Female	255 (38.9)	34 (33.3)	20 (34.5)	185 (40.8)	16 (37.2)	.46
Race/ethnicity						
American Indian and Alaska Native	6 (0.9)	0	0	5 (1.1)	1 (2.3)	.34
Asian	10 (1.5)	0	3 (5.2)	6 (1.3)	1 (2.3)	
Black or African American	4 (0.6)	0	0	3 (0.7)	1 (2.3)	
Native Hawaiian and other Pacific Islander	6 (0.9)	1 (1.0)	0	4 (0.9)	1 (2.3)	
White	599 (91.3)	96 (94.1)	50 (86.2)	416 (91.8)	37 (86.0)	
Medical history						
Hypertension	495 (75.5)	85 (83.3)	49 (84.5)	328 (72.4)	33 (76.7)	.04
Diabetes	197 (30.0)	37 (36.3)	18 (31.0)	131 (28.9)	11 (25.6)	.46
Prior myocardial infarction	178 (27.1)	28 (27.5)	17 (29.3)	122 (26.9)	11 (25.6)	.98
Congestive heart failure	237 (36.1)	55 (53.9)	22 (37.9)	144 (31.8)	16 (37.2)	<.001
Prior stroke	152 (23.2)	16 (15.7)	16 (27.6)	112 (24.7)	8 (18.6)	.18
Pulmonary disease	205 (31.2)	37 (36.3)	13 (22.4)	136 (30.0)	19 (44.2)	.07
Prior direct current cardioversion	171 (26.1)	33 (32.4)	22 (37.9)	107 (23.6)	9 (20.9)	.04
Prior ablation	202 (30.8)	23 (22.5)	12 (20.7)	159 (35.1)	8 (18.6)	.005
CHA_2_DS_2_-VASc Score, mean (SD)^c^	3.60 (2.02)	3.80 (2.05)	4.17 (1.91)	3.48 (2.01)	3.58 (2.13)	.07
ECG						
Ventricular rate on ECG, mean (SD)	75.21 (19.01)	90.96 (18.77)	94.45 (24.23)	69.63 (13.95)	70.72 (20.67)	<.001
Atrial						
Fibrillation	135 (20.6)	90 (88.2)	45 (77.6)	NA	NA	NA
Flutter	23 (3.5)	12 (11.8)	11 (19.0)	NA	NA	NA
Tachycardia	2 (0.3)	0	2 (3.4)	NA	NA	NA
Toronto Atrial Fibrillation Severity Scale score, mean (SD)	8.95 (7.39)	12.70 (8.45)	6.86 (5.47)	8.12 (6.95)	12.23 (8.17)	<.001
Left ventricular ejection fraction, mean (SD)	59.36 (10.11)	56.70 (10.73)	58.03 (9.28)	60.29 (9.52)	57.60 (15.54)	.13
Active medications						
β-blocker	251 (38.3)	64 (62.7)	25 (43.1)	146 (32.2)	16 (37.2)	<.001
Non–dihydropyridine calcium channel blocker	90 (13.7)	20 (19.6)	8 (13.8)	55 (12.1)	7 (16.3)	.24
Any antiarrhythmic drug	171 (26.1)	33 (32.4)	12 (20.7)	116 (25.6)	10 (23.3)	.36
Class IC	71 (10.8)	13 (12.7)	5 (8.6)	49 (10.8)	4 (9.3)	.85
Class III	45 (6.9)	7 (6.9)	4 (6.9)	30 (6.6)	4 (9.3)	.93
Amiodarone	49 (7.5)	13 (12.7)	4 (6.9)	28 (6.2)	4 (9.3)	.14

Abbreviations: AT/AF, atrial tachycardia/atrial fibrillation; ECG, electrocardiogram; NA, not applicable.

^a^ Baseline characteristics, comorbidities, admission data, and laboratory studies, stratified by rhythm self-identification.

^b^ *P* values reflect 4-way comparisons.

^c^ The CHA_2_DS_2_-VASc Score assigns 1 point each for age 65 to 74 years; female sex; and history of heart failure, hypertension, vascular disease (peripheral vascular disease or ischemic heart disease), and diabetes; and 2 points each for age 75 years or older and history of stroke or transient ischemic attack.

Among 160 patients in arrhythmia, patients’ own assessment of rhythm was 64% sensitive for detecting it (102 of 160 patients). On the basis of ECG, 496 patients were in sinus rhythm and demonstrated an accuracy of 91% for detecting it (453 correctly indicated no arrhythmia). Overall, 85% of patients responded consistently with their ECG, including patients with normal rhythm and those in atrial arrhythmia (positive predictive value, 70%; negative predictive value, 89%). Sensitivities for rhythm self-identification, by subgroups of interest, are shown in the Figure.

**Figure.**
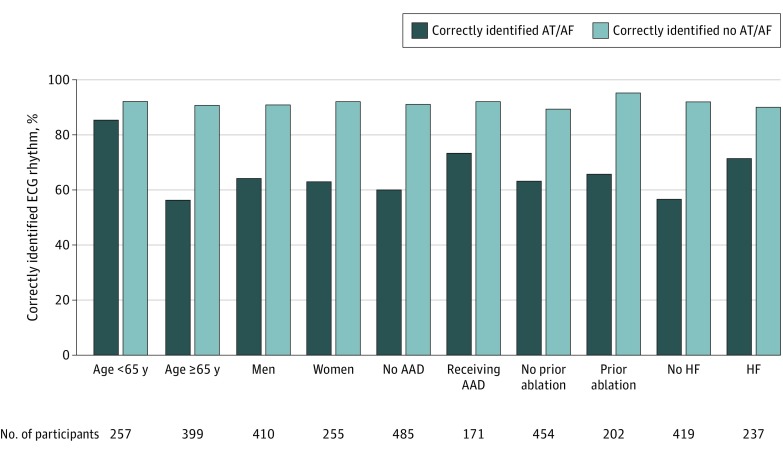
Sensitivity of Correct Rhythm Identification, by Subgroups of Interest AAD indicates antiarrhythmic drug; AT/AF, atrial tachycardia/atrial fibrillation; ECG, electrocardiogram; HF, heart failure.

The mean (SD) overall AFSS symptom score was 8.95 (7.4), with 16% missing. The AFSS symptom score was higher for those who believed they were currently in atrial arrhythmia (mean [SD], 12.70 [8.45] for those who correctly identified AF and 12.23 [8.17] for those in sinus rhythm who incorrectly thought they were in AF) compared with those who did not believe they were in atrial arrhythmia (mean [SD], 8.12 [6.95] for those who correctly identified that they were not in AF and 6.86 [5.47] for those who were in AF but incorrectly thought they were not). In sum, the AFSS symptom score was lowest in the population who were in atrial arrhythmia but were unaware of it.

## Discussion

Our analysis of patient self-identification of arrhythmia yields several important observations that may affect treatment decisions for patients with AF. First, the sensitivity of patient identification for atrial arrhythmia is low; only two-thirds (64%) of patients in atrial arrhythmia correctly identified it. Second, there are important subgroup differences regarding self-identification of cardiac rhythm; younger patients and those receiving an antiarrhythmic drug appear particularly sensitive to detecting atrial arrhythmia. Finally, AFSS symptom score measuring perceived AF symptoms appears to track more closely with perceived vs actual arrhythmia. There are few data available regarding a patient’s ability to correctly identify symptoms of AF, yet we rely on this information nearly every day for clinical decision-making. Naturally, this analysis is limited by the real-world uncontrolled setting, and we were not present while patients answered patient-reported outcomes questions. Regardless, these findings have important implications for both routine, symptomatic management of AF, as well as clinical trials with symptom-driven outcomes. Our data highlight the limitations of relying solely on patient symptom self-reporting for arrhythmia burden and impact and support the routine use of confirmatory testing to diagnose AF and to evaluate the association of symptoms with arrhythmia.
